# Study on the analgesic and anti-inflammatory mechanisms of Jingu Zhitong gel in the treatment of knee osteoarthritis through the IL-17, NGF-TrkA, and COX-2/PGE2 pathways

**DOI:** 10.3389/fphar.2026.1791915

**Published:** 2026-04-17

**Authors:** Lu Li, Yiyao Liang, Jing Dong, Zhenzhen Su, Xinyu Xu, Ziyin Wu, Xinzhuang Zhang, Jifeng Li, Zhenzhong Wang, Wenjun Liu, Liang Cao, Wei Xiao

**Affiliations:** 1 State Key Laboratory of Technologies for Chinese Medicine Pharmaceutical Process Control and Intelligent Manufacture (Jiangsu Kanion Pharmaceutical Co., Ltd.), Nanjing, China; 2 School of Pharmacy, Shanghai University of Traditional Chinese Medicine, Shanghai, China; 3 Jiangsu Kangyuan sunshine Pharmaceutical Co., Ltd., Nanjing, China

**Keywords:** COX-2/PGE2 pathway, IL-17 signaling pathway, Jingu Zhitong gel (JGZTG), knee osteoarthritis (KOA), NGF-TrkA pathway, transcriptomics

## Abstract

**Introduction:**

Knee osteoarthritis (KOA) is a degenerative bone and joint disease. Jingu Zhitong Gel (JGZTG) exhibits a promising therapeutic effect in the clinical treatment of knee osteoarthritis (KOA). However, the mechanism of JGZTG in treating KOA remains unclear.

**Methods:**

We established a rat model of KOA through surgery and treated rats with JGZTG for 21 days. The pain threshold was measured by the Ugo Basile joint pain tester, the weight bearing on the right foot was detected by a bipedal balance tester, the inflammatory factor levels were measured by ELISA kits, and the joint morphology score was evaluated. Hematoxylin and eosin staining (H&E) was used to evaluate the pathological lesions, while toluidine blue staining was used to observe the proteoglycan depletion. Transcriptomic analysis was conducted to elucidate the potential mechanisms of JGZTG in treating KOA. Real-time quantitative Polymerase Chain Reaction (RT-qPCR), and immunofluorescence (IF) assays were conducted to validate transcriptomic results and investigate the analgesic mechanisms.

**Results:**

After 21 days of treatment, JGZTG significantly increased the pain threshold and the weight bearing on the right foot in KOA rats, furthermore, reduced the levels of inflammatory factors (TNF-α, IL-6, IL-1β, and PGE2) in the serum and joint lavage fluid. In addition, JGZTG significantly decreased the joint morphology score, reduced synovial damage, alleviated cartilage injury, and reduced proteoglycan depletion in KOA models. The results of transcriptomic analysis showed that the anti-inflammatory effect of JGZTG in treating KOA was associated with the IL-17 signaling pathway. Further validation revealed that the relative mRNA and protein expression of IL-17RB, FosB, MMP1b, MMP3, MMP13, CCL17, and CXCL6 were regulated by JGZTG. In addition, JGZTG can reduce the mRNA and protein overexpression of NGF, Ntrk1, Trpv1, Ptgs2, PGE2, EP4, TAC1, and Calca, which are the key factors of the NGF-TrkA and COX-2/PGE2 pathways.

**Conclusion:**

JGZTG exhibits pain-relieving and anti-inflammatory effects in KOA, which were linked to altered IL-17, NGF-TrkA, and COX-2/PGE2 pathways. These findings have provided experimental evidence for the mechanisms underlying the therapeutic effects of JGZTG in KOA.

## 1 Introduction

Knee osteoarthritis (KOA) is a degenerative bone and joint disease, which is more common in middle-aged and elderly people, with knee joint swelling, pain, stiffness, mobility disorders, and muscle atrophy as the main symptoms in clinical practice (Dantas et al., 2021). Studies have shown that with the aging of the population, 250 million people worldwide are currently suffering from KOA, which has become a severe public health problem and has brought economic burden to both patients and society (Hunter and Bierma-Zeinstra, 2019). The major pathological hallmarks of KOA are synovial inflammation, articular surface cartilage degeneration, meniscal degeneration, ligament and muscle pathologic changes, subchondral sclerosis, and osteophyte hyperplasia, etc (Zhou et al., 2023). Although the pathogenesis of KOA has not been fully understood, joint inflammation may play an important role in the onset of pain in KOA, and aggravates cartilage damage and eventually evolves into chronic pain (Ashraf et al., 2018; Ostojic et al., 2019). Antipyretic analgesics, nonsteroidal anti-inflammatory drugs (NSAIDs), or intra-articular corticosteroids are commonly used in the treatment of KOA in clinical practice; however, long-term use of these drugs may cause side effects such as severe gastrointestinal reactions and cannot reverse disease progression (Liu et al., 2025). Therefore, it is urgent to find new treatment methods in Chinese medicine.

Jingu Zhitong Gel (JGZTG) (Approval number: Z20200003, patent number: CN101288711B), which was formerly known as “Tongning Gel” prior to its market launch, shares the same prescription composition and preparation methods. JGZTG is a formula containing twelve Chinese medicine, exhibits a promising therapeutic effect in the clinical treatment of KOA. The compositions and proportion of JGZTG were listed in Table 1. According to traditional Chinese medicine (TCM) theory, JGZTG is used for kidney deficiency, tendon and pulse stasis symptoms with the function of promoting blood circulation and regulating qi, dispelling wind and dampness, unblocking collaterals, and relieving pain (Lv et al., 2024). The clinical experience showed that JGZTG could significantly reduce the pain index, total score, stiffness score, and joint motion score according to the Western Ontario and McMaster Universities Osteoarthritis Index (WOMAC), and the TCM syndrome score of KOA patients (Liu, 2012; Zhao et al., 2020). The systematic investigations of the chemical constituents in JGZTG have been conducted using Gas Chromatograph-Mass Spectrometer (GC-MS) and Ultra-performance liquid chromatography coupled with quadrupole-time of flight mass/mass spectrometry (UPLC-Q-TOF-MS/MS) (Fu et al., 2020; Lv et al., 2024). However, the mechanism of JGZTG in treating KOA remains unclear. Our previous research demonstrated that JGZTG may inhibit the progress of synovitis by regulating the expression of Interleukin-1 beta (IL-1β), Prostaglandin E2 (PGE2), and NLRP3 (NOD-like receptor family pyrin domain containing 3) in rats with KOA (Peng et al., 2023).

**TABLE 1:** Composition and proportion of JGZTG.

Chinese name	Botanical name	Proportion (g)	Category
Yanhusuo	*Çorydalis yanhusuo* (Y. H. Chou & Chun C. Hsu) W. T. Wang ex Z. Y. Su and C. Y. Wu	107.5	Chief
Chuɑnxiong	*Ligusticum chuanxiong* S. H. Qiu, Y. Q. zeng, K. Y. Pan, Y. C. Tang and J. M. Xu	86	Deputy
Weilingxiɑn	*Clematis chinensis* Osbeck	86	Deputy
Shenjincɑo	*Lycopodium japonicum* Thunb.	107.5	Assistant
Tougucao	*Phryma leptostachya* L.	107.5	Assistant
Lulutong	*Liquidambar formosana* Hance	86	Assistant
Haitongpi	*Erythrina variegata* L.	107.5	Assistant
Fangfeng	*Saposhnikovia divaricata* (Turcz.)Schischk.	107.5	Assistant
Huɑjiɑo	*Zanthoxylum bungeanum* Maxim.	107.5	Assistant
Bohe	L-Menthol	3.6	Assistant
Bingpian	*Borneolum Syntheticum*	7.2	Assistant
Niuxi	*Achyranthes bidentata* Blume	86	Envoy

Therefore, we established KOA model rats through surgery to investigate the therapeutic effect of JGZTG (Li et al., 2024; Zhang et al., 2025). Furthermore, transcriptomic analysis was used to evaluate the potential mechanism of JGZTG in the treatment of KOA. The interleukin (IL) −17 signaling pathway, associated with synovial inflammation, is the most significant pathway in KOA through integrated bioinformatics analysis (Xu et al., 2024). IL-17 amplifies the synovial inflammatory and cartilage damage effects in KOA by promoting the expression of pro-inflammatory factors, chemokines, and matrix metalloproteinases (Xiao et al., 2023). The nerve growth factor (NGF) -tyrosine kinase receptor A (TrkA) pathway serves as a crucial pain transmission pathway in KOA. Research has found that augmentation of NGF-TrkA signaling in the joint synovium and the peripheral sensory neurons facilitates pro-nociception and centralized sensitization, thereby aggravating joint pain (InSung et al., 2022). Cyclooxygenase-2 (COX-2) inhibitors are used in the treatment of osteoarthritis; inhibition of COX-2 reduces the production of prostaglandins, which decreases inflammation and pain (Richard et al., 2023). Real-time quantitative Polymerase Chain Reaction (RT-qPCR)**,** western blot (WB), and immunofluorescence (IF) techniques were used to validate the mechanism of how JGZTG affects KOA through IL-17, NGF-TrkA, and COX-2/PGE2 pathways. In summary, JGZTG exhibits a multi-pathway regulatory mechanism in KOA, which could provide a new option for KOA treatment.

## 2 Materials and methods

### 2.1 Materials

Diclofenac Diethylamine Gel (DDE) was purchased from GSK Consumer Healthcare, and JGZTG was obtained from Jiangsu Kanion Pharmaceutical Co., Ltd. The ELISA kits for IL-1β, TNF-α, IL-6, and PGE2 were purchased from MultiSciences (LiankeBio) (Hangzhou, China).

Rabbit IL-17RB antibody, Rabbit MMP13 antibody, Rabbit MMP3 antibody, Rabbit MMP13 antibody, and GCP2 (CXCL6) Ab were purchased from Affinity (Cincinnati, United States). FosB (5G4) Rabbit mAb was purchased from CST (Massachusetts, United States), NGF Rabbit pAb was purchased from Immunoway (Texas, United States), and TARC (CCL17) Polyclonal Antibody was purchased from Invitrogen (Carlsbad, United States). The above antibodies were used in Western blots.

Calca, NGF, Ptgs2, and TAC1 antibodies were purchased from Servicebio (Wuhan, China); Ntrk1, PGE2, EP4, and TRPV1 antibodies were purchased from ABclonal (Wuhan, China). The above antibodies were used in Immunofluorescence.

### 2.2 Preparation of JGZTG

JGZTG was supplied by Jiangsu Kanion Pharmaceutical Co., Ltd. (Jiangsu, China). Table 1 displays the composition and proportion of JGZTG. JGZTG was prepared by first subjecting *Zanthoxylum bungeanum* Maxim., *Ligusticum chuanxiong* S. H. Qiu, Y. Q. Zeng, K. Y. Pan, Y .C. Tang and J. M. Xu, and *Saposhnikovia divaricata* (Turcz.) Schischk. to hydro-distillation with 10-fold water for 8 h. The volatile oil was collected, and the herb residue was decocted with 10-fold water for 1 h, then combined with the distilled aqueous solution and filtered. Separately, *Çorydalis yanhusuo* (Y. H. Chou and Chun C. Hsu) W. T. Wang ex Z. Y. Su and C. Y. Wu, *Lycopodium japonicum* Thunb., *Phryma leptostachya* L*., Liquidambar formosana* Hance, *Erythrina variegata* L., *Achyranthes bidentata* Blume, and *Clematis chinensis* Osbeck was reflux-extracted twice with 10-fold 70% ethanol for 1 h. The combined ethanol extracts were filtered, concentrated to recycle ethanol, and then mixed with the aqueous filtrate. The combined extract was concentrated to a relative density of 1.20–1.25 (25 °C). Finally, The gel was prepared by fully swelling carbomer in distilled water, sequentially incorporating triethanolamine and glycerol, followed by a *Borneolum Syntheticum* and L-Menthol eutectic mixture, the volatile oil, the concentrated extract, and additional water, then triturating to homogeneity before packaging.

### 2.3 Animals’ treatment

Male Sprague-Dawley rats weighing 280–300 g were purchased from Jiangsu Huachuang Xinnuo Pharmaceutical Technology Co. Ltd. (animal certificate number: SCXK (SU) 2020-0009). The rats were housed under a room temperature of 24 °C–26 °C, a relative humidity of 55%, and a 12-h light-dark cycle. The experiments were approved by the Animal Care and Use Committee of China Pharmaceutical University (Animal Ethics Approval Number: CPU2023-09-018).

After 1 week of adaptive feeding, the medial collateral ligament and anterior cruciate ligament of the rats’ right knee joint were transected, and the medial meniscus was removed. Antibiotics were injected intramuscularly for 3 days to prevent infection, and the rats were inspected to exercise for 0.5 h every day for 4 weeks to establish the KOA model. In the sham group, the medial skin of the rats’ right knee joint was just incised and sutured.

After 4 weeks of modeling, the pain threshold of the rats was measured by the Ugo Basile joint pain tester, and the rats were randomly grouped into six groups according to the pain threshold: the sham group (Sham, *n* = 18), the model group (Model, *n* = 18), Diclofenac Diethylamine Cream group (DDE, *n* = 18, 0.2 g/kg), JGZTG low dosage group (JGL, *n* = 18, 0.6 g/kg), JGZTG middle dosage group (JGM, *n* = 18, 1.2 g/kg), JGZTG high dosage group (JGH, *n* = 18, 2.4 g/kg). The drugs were applied to the knee joint skin surface twice a day, lasting for a total of 21 days.

### 2.4 Pain symptom detection

12 rats were randomly selected from each group. 4 h after the final drug administration, the pain threshold of rats was measured by the Ugo Basile joint pain tester, and the weight bearing on the right foot of rats was measured by a bipedal balance tester.

### 2.5 Sample collection

24 h after the final drug administration, the orbital blood of rats was collected, and the serum was separated. Besides, rats in each group were anaesthetized and dissected, and the knee joint, joint lavage fluid, synovium, and L4-L6 DRG of the spinal cord were obtained.

### 2.6 Enzyme-linked immunosorbent assay (ELISA)

The levels of inflammatory factors IL-1β, IL-6, PGE2, and Tumor necrosis factor-α (TNF-α) in rats serum and joint lavage fluid were measured by ELISA kits. ELISA assays were conducted using samples from *n* = 10 randomly selected animals per group.

### 2.7 Articular cartilage morphology scores evaluation

The knee joints of rats were observed after dissection on 10 randomly selected rats from each group, and the articular cartilage morphological scores were evaluated according to the following principles (Table 2).

**TABLE 2:** Articular cartilage morphology scores evaluation.

Score	Assess symptoms
0	The articular surface was smooth, and the color was normal
1	The articular surface was rough, with small fissures and grey in color
2	The articular surface was eroded, and the cartilage was moderately injured
3	The articular surface was ulcerous, and the cartilage was deeply damaged
4	The cartilage was exfoliated, and the subchondral bone was exposed

### 2.8 Hematoxylin and eosin and toluidine blue staining

H&E staining was conducted on synovial tissue (*n* = 6) and on articular cartilage from a random subset of rats (*n* = 10) for knee joint histological assessment. Cartilage degradation was semi-quantitatively assessed using a modified Wakitani histological scoring system (Orth and Madry, 2015). This system evaluates four distinct histopathological dimensions of osteoarthritis-like lesions (Table 3 **)**. And the total score ranges from 0 to 9, with lower scores indicating better-preserved cartilage architecture and milder degeneration. Histological scoring was performed by a single investigator who was blinded to the experimental groups.

**TABLE 3:** Articular cartilage histological scores evaluation.

Histological parameter	Score	Description
Articular cartilage structure	0	Hyaline cartilage
	1	Predominantly hyaline cartilage
	2	Fibrocartilage
	3	Minimal fibrocartilage
	4	Complete loss of cartilage
Chondrocyte count	0	Normal cellularity
	1	Mild proliferation
	2	Moderate proliferation with clustered chondrocytes
	3	Severe chondrocyte depletion
Osteophyte formation	0	Absent
	1	Present
Epiphyseal plate damage	0	Absent
	1	Present

Toluidine blue staining was used to observe cartilage lesions of rats’ knee joints. Specifically, for the synovial and cartilage tissue block, fixation was achieved with 4% paraformaldehyde for 24 h, followed by a dehydration process involving sequential immersion in ethanol of increasing concentrations. The tissue blocks were subsequently transferred into xylene for clearing and then embedded in paraffin. Utilizing a microtome, continuous sections of 5 μm thickness were meticulously prepared. These tissue sections were sequentially subjected to H&E staining (Yu et al., 2025) and toluidine blue staining. Sections were evaluated and imaged under optical microscopy.

### 2.9 Transcriptomics analysis

RNA extraction, library construction, and transcriptome sequencing were conducted by Hangzhou Lianchuan Biotechnology Co., Ltd. The total RNA of three synovial tissue samples from each group was extracted using TRIzol reagent (Thermo Fisher). The concentration and purity of total RNA were assessed using the 6000 Nano LabChip Kit (Agilent, CA, United States), while the total RNA integrity number (RIN) was analyzed using Bioanalyzer 2100. The synovial tissue RNA samples with RIN > 7.0 were used to construct the sequencing library. Transcriptome sequencing was performed using the Illumina HiSeq NovaSeq 6000 platform (Illumina, United States).

The IL-17 signaling pathway emerges as the most dominant canonical pathway in KOA synovial tissue based on established literature (Xu et al., 2024). We pre-specified a set of candidate genes involved in Interleukin 17 Receptor (IL-17RA, IL-17RB, IL-17RC), Downstream Transcription Factors (FOS, FOSB, JUN, etc.), Pro-Inflammatory Cytokines (IL6, IL1B, TNF, etc.), C-C and C-X-C motif chemokine ligand (CCL2, CCL7, CCL20, CXCL2, CXCL5,etc.), Matrix Metalloproteinases (MMP1b, MMP3, MMP9, MMP9, MMP13), and antimicrobial Peptides. The differentially expressed genes (DEGs) were identified using DESeq2 and edgeR, with the parameters of false discovery rate (FDR) ≤ 0.05 and the absolute fold change (FC) ≥ 2.

To identify pathways potentially modulated by JGZTG, genome-wide differential expression analysis was performed using DESeq2. A Venn diagram was created to depict the intersection of drug-disease targets, and the potential therapeutic genes were further screening for reverse genes—defined as genes significantly upregulated in the Model group versus Sham (FDR ≤ 0.05, log_2_FC ≥ 1) and significantly downregulated in the JGZTG group versus Model (FDR ≤ 0.05, log_2_FC ≤ −1), or *vice versa* for downregulated disease genes. To elucidate the functional roles of the two gene sets, we performed parallel functional enrichment analyses. Specifically, the intersecting genes and the reverse-transcribed genes were separately analyzed for Gene Ontology (GO) term enrichment and Kyoto Encyclopedia of Genes and Genomes (KEGG) pathway annotation using the KOBAS tool (http://bioinfo.org/kobas). No predetermined pathway filters were applied. Statistical significance for the enriched functions and pathways was defined as *P* < 0.05.

All statistical analyses and visualizations, including heatmaps and Venn diagrams, were performed using R (version 4.4.2).

### 2.10 Real-time quantitative polymerase chain reaction

The total RNA of synovial tissue and dorsal root ganglion (DRG) was extracted using the TaKaRa MiniBEST Universal RNA Extraction Kit (9767, Takara, China) and reverse-transcribed into cDNA using PrimeScript™ RT reagent Kit with gDNA Eraser to obtain cDNA (RR047A, Takara, China) for qPCR amplification. qPCR was then performed with TB Green® Premix Ex Taq™ II (Tli RNaseH Plus) (RR820Q, Takara, China) on LightCycler®480 Instrument II (Roche, Switzerland). Primer sequences are listed in Table 4, with β-actin as the reference gene. The relative mRNA levels were calculated using the 2^−ΔΔCT^ method. IL-17RB, FOSB, MMP1b, MMP3, MMP13, CCL17, CXCL6, and NGF were selected as detection indices of synovial tissue, while mRNA levels of NGF, Neurotrophic receptor tyrosine kinase 1 (Ntrk1), Transient receptor potential cation channel subfamily V member 1 (TRPV1), Prostaglandin E receptor 4 (EP4), Tachykinin precursor 1 (TAC1), and Calcitonin-related polypeptide alpha (Calca) in DRG were detected.

**TABLE 4:** Primer sequences.

Gene	Forward primers 5′-3′	Rerverse primers 5′-3′
MMP1b	CTA​AAT​CCC​ACT​TTG​CCC​ACG	TGG​TCC​ACG​TCT​CAT​CCA​GA
MMP3	CCT​CTG​AGT​CTT​TTC​ATG​GAG​GG	TCA​GTG​CGC​CAA​GTT​TCA​GA
FOSB	TCC​CAC​CCC​TGT​GCA​GTA​TT	TTG​GAG​GGT​TGG​GGG​TCA​AT
CCL17	GAC​CTT​CGC​CTG​GAC​TTC​TG	ACT​CTC​GGC​CTA​CAT​TGG​TG
CXCL6	CTT​GAC​CCA​GAA​GCT​CCG​TT	CTT​GCC​TTC​CCT​GGG​TAC​AG
IL17RB	CTGGTGCTGGTGGCTAC	GAT​GTC​TTT​GTG​CTC​CTT​CCT​TGC
MMP13	TGC​TGC​ATA​CGA​GCA​TCC​AT	CCC​CGT​GTC​CTC​AAA​GTG​AA
NGF	CGAAGGGGAGCGCATCG	GCC​GAT​CAA​AAA​CGC​TGT​GA
TRPV1	CAT​GCT​TCT​CGT​GGA​ACC​CT	CCC​CAA​CGG​TGT​TTT​TCA​GC
TAC1	GTC​CGA​CCG​CAA​AAT​CCA​AC	AAG​ATG​CTC​AAA​GGG​CTC​CG
Ntrk1	GCT​GCC​TTT​ATG​GAC​AAC​CC	TGG​GTC​TCT​TGA​TGT​GCT​GT
Calca	CCA​GAT​CAA​GAG​TCA​CCG​CC	CCT​CCC​TGA​GCA​GGA​ACC​TC
β-actin	CTG​TGT​GGA​TTG​GTG​GCT​CT	AGC​TCA​GTA​ACA​GTC​CGC​CT

### 2.11 Western blot

Three synovial tissue samples of each group were selected and homogenized with the IP lysis buffer (Genomeditech). Then, the protein concentration was determined using the BCA kit (E112-02, Vazyme). The protein samples were mixed with loading buffer (139,221, Genomeditech) after dilution, and boiled at 100 °C for 10 min to denature. Proteins were separated using SDS-PAGE (C641110, Sangon, China) and transferred to polyvinylidene difluoride membrane (PVDF) (FFP39, Beyotime). After washing with TBST, the membranes were blocked with 5% skim milk powder and then incubated with primary antibodies overnight at 4 °C. Subsequently, the membranes were washed three times with TBST and incubated with secondary antibodies for 2 h. After three additional washes, the ECL reagent was applied to capture the blots, and the membranes were scanned with an automated gel imaging system (4600SF, Tanon, China). The band intensities were analyzed using ImageJ software.

The following antibodies were used: MMP13 Antibody (AF5355, Affinity), IL17RB Antibody (DF2510, Affinity), MMP3 Antibody (AF0217, Affinity), GCP2 Antibody (DF13470, Affinity), FosB (5G4) Rabbit mAb (2251, Cell Signaling), TARC Polyclonal Antibody (PA5-34515, Invitrogen), NGF Rabbit pAb (YT3114, Immunoway), Anti-β-Actin Antibody (GM-34500AB, Invitrogen). β-actin was selected as loading control.

### 2.12 Immunofluorescence

The DRG slices were deparaffinized using a dewaxing agent and anhydrous ethanol, followed by antigen retrieval. The primary antibodies targeting NGF, Ntrk1, TRPV1, Prostaglandin-endoperoxide synthase (Ptgs2), PGE2, EP4, TAC1, and Calca were added after blocking with BSA and incubated at 4 °C overnight. Following 3 PBS rinses, slices were incubated with the secondary antibody at room temperature. Then, PBS wash was performed three times, and DAPI staining solution was added, which was further incubated at room temperature. A fluorescence quencher was added after PBS washes. Subsequently, the plates were encapsulated with an anti-fluorescence quenching mounting agent, and images were captured with a fluorescence microscope (Nikon) and a Pannoramic scanner (3DHistech). The red positive cells (%) were quantified with Aipathwell software.

### 2.13 Data processing and statistical analysis

All data were displayed as means ± standard deviation (SD). Statistical analyses were performed with GraphPad Prism 9.5 software (San Diego, CA, United States). Two-way analysis of variance (ANOVA) was used to compare data across groups, and statistical significance was defined as *P* < 0.05.

## 3 Results

### 3.1 JGZTG improves the pain symptoms in KOA rats

**FIGURE 1:**
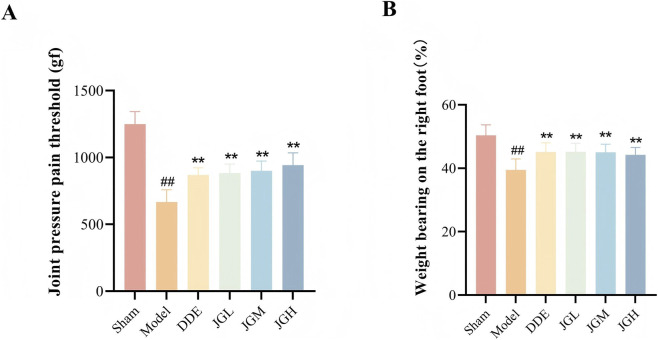
JGZTG improves the pain symptoms of KOA rats. **(A)** Joint pressure pain threshold (*n* = 12); **(B)** weight bearing on the right foot (*n* = 12). Data are presented as the means ± SD. ^##^ *P* < 0.01 vs. Sham; ^**^ *P* < 0.01 vs. Model.

The joint pressure pain threshold and weight bearing on the right foot were measured in KOA rats to estimate the analgesic effect of JGZTG. The results showed a significant decrease in joint pressure pain threshold and weight bearing on the right foot in the model group compared with the sham group (Figure 1, ^##^ *P* < 0.01). Compared with the model group, the joint pressure pain threshold and weight bearing on the right foot of the three JGZTG dose groups could be increased; furthermore, JGZTG improved the joint pressure pain threshold in a dose-dependent way (Figure 1, ^*^ *P* < 0.05, ^**^ *P* < 0.01). These results indicate that JGZTG has an analgesic effect after 21 days of treatment, suggesting that JGZTG plays an important role in the chronic pain of KOA.

### 3.2 JGZTG attenuates the inflammatory response in KOA rats

Cytokines participate in the pathogenesis of KOA (Du et al., 2023). IL-1β and TNF-α can be induced through autocrine mechanisms, while also stimulating the generation of other pro-inflammatory cytokines (IL-6) (Koyama et al., 2021; Sanchez-Lopez et al., 2022) and inflammatory mediators (PGE2). As a mediator of inflammatory pain (Gahbauer et al., 2023), PGE2 can induce pain sensitisation (Kimourtzis et al., 2024), inhibit cartilage synthesis, promote cartilage degradation (Wang et al., 2025), and accelerate disease progression of OA (Yang et al., 2024).

**FIGURE 2:**
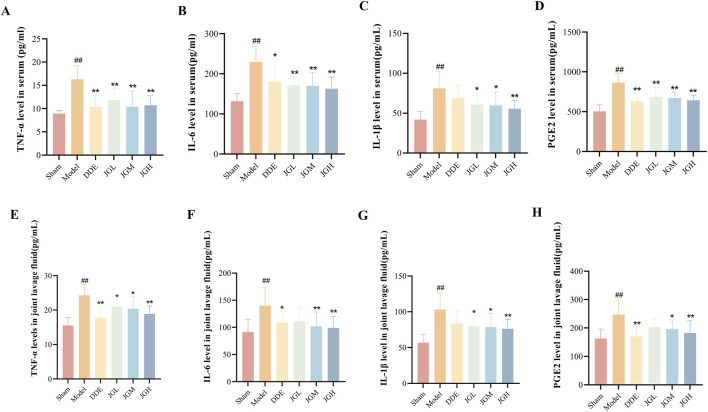
JGZTG ameliorates the inflammation by reducing the levels in KOA rats. The levels of **(A)** TNF-α, **(B)** IL-6, **(C)** IL-1β, and **(D)** PGE2 in serum of KOA rats are measured by ELISA (n = 10). The levels of **(E)** TNF-α, **(F)** IL-6, **(G)** IL-1β, and **(H)** PGE2 in joint lavage fluid of KOA rats are measured by ELISA (n = 10). Data are presented as the mean ± SD. ^##^ *P* < 0.01 vs. Sham; ^**^ *P* < 0.01 vs. Model; ^*^ *P* < 0.05 vs. Model.

Therefore, the levels of inflammatory factors were detected in all groups of rats. The results showed significantly elevated levels of TNF-α, IL-6, IL-1β, and PGE2 in both serum and joint lavage fluid of the model group compared with the sham group (Figures 2A–H, ^##^ *P* < 0.01). After 21 days of JGZTG treatment, TNF-α, IL-6, IL-1β, and PGE2 levels in serum and joint lavage fluid were decreased in a concentration-dependent manner compared with the model group (Figures 2A–H, ^*^ *P* < 0.05, ^**^ *P* < 0.01). These results indicate that JGZTG alleviates joint inflammation and relieves joint pain by inhibiting the release of pro-inflammatory cytokines TNF-α, IL-6, IL-1β, and the inflammatory pain modulator PGE2.

### 3.3 JGZTG improves joint injury in KOA rats

**FIGURE 3:**
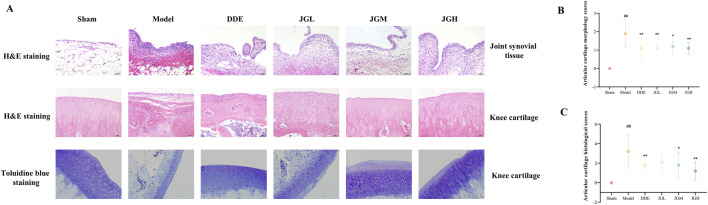
JGZTG improves joint injury in KOA rats. H&E staining representative images of **(A)** joint synovial tissue sections and knee cartilage sections in rats (200×); toluidine blue staining representative images of knee cartilage sections in rats (200×). Data are presented as the means ± SD. ^##^ *P* < 0.01 vs. Sham; ^**^ *P* < 0.01 vs. Model; ^*^ *P* < 0.05 vs. Model. **(B)** The articular cartilage morphology scores (assessed by gross observation prior to histology) in rats (n = 10). **(C)** The articular cartilage histological scores in rats (n = 10).

The observation results of rat’ knee joint tissue showed that the knee joint surface of the sham group was smooth and the color was normal, but the knee articular surface of the model group was rough and eroded, and the cartilage was injured. Consequently, the articular cartilage morphology score in the model group was significantly increased compared with the sham group (Figure 3B, ^##^ *P* < 0.01). Interestingly, JGZTG could alleviate the damage of articular cartilage surface morphology and decrease the articular cartilage morphology scores compared with the model group (Figure 3B, ^*^ *P* < 0.05, ^**^ *P* < 0.01).

H&E staining results of joint synovial tissue sections showed that the joint synovial tissue of the sham group was covered by 2–4 layers of synovial cuboidal cells neatly, and the sub-synovial tissue consisted of loose connective fat tissue, with a dense connective tissue layer outside. Besides, there was no edema, inflammatory cell infiltration, or fibrosis observed in the synovial tissue of the sham group. The synovial tissue structure of the KOA model rats was similar to that of the sham group, but mild to severe degeneration and necrosis of synovial cells could be observed, with some showing no obvious synovial cells. There was mild or moderate inflammatory cell infiltration in the synovial tissue, and the inflammatory cells were mainly mononuclear. Most of the synovial tissues varied in density, with some showing abundant dense connective tissue. However, three dose groups of JGZTG could attenuate the pathological injury of joint synovial tissue in KOA rats (Figure 3A).

H&E staining results of knee cartilage sections showed that the knee joint of rats in the sham group was covered by transparent cartilage, with small, single cells on the surface. The structures of epiphyseal bone tissue and epiphyseal growth plate tissue were normal without fibrous tissue hyperplasia. In the model group, the knee cartilage cells were degenerated, the cartilage tissue thickness was varied in thickness, the cartilage layer was missing or hyperplastic, and the cartilage surface was covered by fibrous tissue. The trabecular bone of the epiphysis was destroyed, and the focal fibrous tissue was hyperplastic. The growth plates of the epiphyseal plate were disordered, with different thickness and irregular shape. The pathological injury of articular cartilage in rats with KOA was obviously improved following treatment with JGZTG at three different doses (Figure 3A). Accordingly, the articular cartilage histological score in the model group was significantly increased compared with the sham group (Figure 3C, ^##^ *P* < 0.01). Notably, JGZTG could alleviate the damage of articular cartilage and decrease the articular cartilage histological scores compared with the model group (Figure 3C, ^*^ *P* < 0.05, ^**^ *P* < 0.01).

Toluidine blue staining of knee cartilage sections showed that the knee joint surface of the sham group was exhibited intense metachromatic (blue-purple) staining, while the blue-purple area of the model group was significantly reduced. Moreover, the treatment with JGZTG could alleviate the reduction of blue-purple staining area in the model group, suggesting that JGZTG could inhibit the proteoglycan depletion (Figure 3A).

### 3.4 Analysis of related genes and pathways by transcriptomics

**FIGURE 4:**
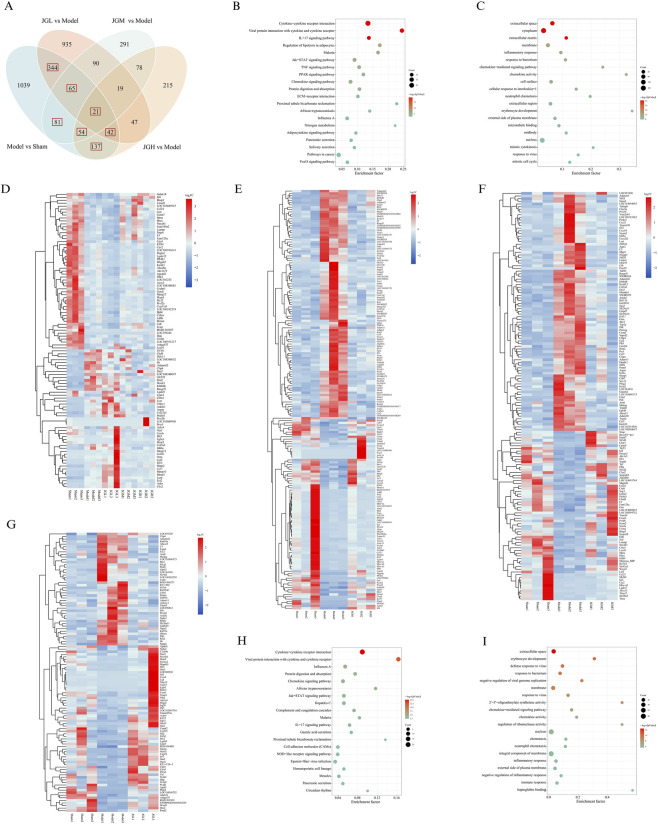
Transcriptomic analysis of synovial tissues in the KOA model and treating with JGZTG. **(A)** Venn diagram illustrating the overlapping DEGs. **(B)** Bubble chart showing the top 20 significantly enriched GO terms of 744 overlapping DEGs. **(C)** Bubble chart presenting the top 20 enriched KEGG pathways of 744 overlapping DEGs. **(D)** Heatmap displaying expression patterns of the top 100 candidate target genes among 744 overlapping DEGs. **(E)** Heatmap showing that 110 genes in JGL met the reversal criteria. **(F)** Heatmap showing that 184 genes in JGM met the reversal criteria. **(G)** Heatmap showing that 194 genes in JGH met the reversal criteria. **(H)** Bubble chart presenting the top 20 enriched KEGG pathways of 358 genes reversed by JGZTG. **(I)** Bubble chart showing the top 20 significantly enriched GO terms of 358 genes reversed by JGZTG.

To further investigate the mechanisms underlying the effects of JGZTG on KOA, transcriptomic and bioinformatic analyses were performed. Compared with the Sham group, a total of 1783 differentially expressed genes (DEGs) were identified from the Model group. A Venn diagram was used to analyze the interacting DEGs between the Sham and Model groups, as well as between the Model group and different JGZTG dose groups. A total of 744 overlapping DEGs were identified, which might be the key DEGs for JGZTG to treat KOA. Heatmap analysis across all dose groups identified 358 genes meeting the reversal criteria: 110 in the JGL, 184 in the JGM, and 194 in the in the JGH (Figures 4E–G). Next, to identify functionally relevant pathways, we performed GO and KEGG enrichment analyses on the 744 overlapping DEGs and the 358 genes reversed by JGZTG. GO enrichment analysis of both gene sets indicated that JGZTG affects biological processes (e.g., inflammatory response, chemokine-mediated signaling pathway), cellular components (e.g., extracellular space), and molecular functions (e.g., chemokine activity) in the KOA rat model (Figures 4C,I). KEGG pathway analysis revealed significant enrichment in pathways including cytokine-cytokine receptor interaction, viral protein interaction with cytokines and cytokine receptors, as well as the IL-17, Jak-STAT, and chemokine signaling pathways (Figures 4B,H). Consistent with these findings, Further heatmap analysis of the overlapping DEGs demonstrated that JGZTG treatment reversed the majority of expression changes induced by the KOA surgical model (Figure 4D). Based on these transcriptomic findings, the IL-17 signaling pathway was selected for subsequent experimental validation.

### 3.5 JGZTG treatment ameliorates the inflammatory reaction of KOA: involvement of the IL-17 pathway

**FIGURE 5:**
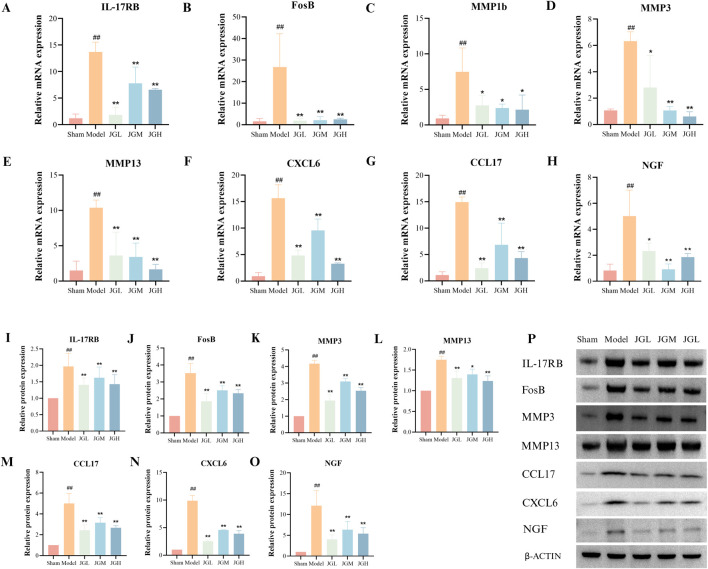
JGZTG inhibits synovial inflammation in KOA rats. **(A–H)** RT-qPCR results of IL-17RB, FosB, MMP1b, MMP3, MMP13, CCL17, CXCL6 and NGF mRNA levels in the synovial tissue (n = 3). **(P)** WB results of IL-17RB, FosB, MMP3, MMP13, CCL17, CXCL6, and NGF proteins in the synovial tissue, and **(I–O)** quantitative analysis results of protein levels (n = 3). Data are presented as the means ± SD. ^##^ *P* < 0.01 vs. Sham; ^**^ *P* < 0.01 vs. Model; ^*^ *P* < 0.05 vs. Model.

To explore how JGZTG affects the inflammatory reaction of KOA, we performed RT-qPCR and WB experiments to analyze the levels of key indicators involved in IL-17 pathways in the synovial tissue of each group. The RT-qPCR results revealed that the relative expression of IL-17RB, FosB, MMP1b, MMP3, MMP13, CCL17, and CXCL6 mRNA in synovial tissue of the model group were significantly increased compared with the sham group (^##^ *P* < 0.01). As an upstream receptor in the IL-17 signaling pathway, activation of IL-17RB directly stimulates Activator protein-1 (AP-1) (with FOSB as its core component), thereby influencing the downstream expression of MMP1b, MMP3, MMP13, CCL17, and CXCL6. Moreover, the relative expressions of these mRNA were significantly decreased after treatment in all JGZTG dose groupst compared with the model group (^**^ *P* < 0.01, ^*^ *P* < 0.05) (Figures 5A–G). WB results showed a significant increase in protein expression of IL-17RB, FosB, MMP3, MMP13, CCL17, and CXCL6 in the synovial tissue of the KOA rats compared with the sham group (^##^ *P* < 0.01), which was significantly reversed by JGZTG treatment compared with the model group (^**^ *P* < 0.01, ^*^ *P* < 0.05) (Figures 5I–P). The above findings suggest that the mechanism of JGZTG protecting KOA rats from inflammatory response may be achieved via regulating IL-17 signaling pathway, which is consistent with the transcriptomics findings.

Inflammatory mediators released via the IL-17 signaling pathway, such as IL-1β and TNF-α, can directly stimulate an increase in NGF release. NGF is considered closely related to inflammatory pain and may serve as a potential target for pain treatments (Liu et al., 2024). Experimental research has found that the mRNA and protein relative expression of NGF in the synovial tissue of the model group was significantly increased compared with the sham group (^##^ *P* < 0.01) (Figure 5H). Following treatment with different doses of JGZTG, the mRNA and protein relative expression of NGF was significantly decreased compared with the model group (^**^ *P* < 0.01, ^*^ *P* < 0.05) (Figure 5). It is hypothesized that JGZTG can alleviate KOA pain symptoms potentially through suppression of NGF-related pathways.

### 3.6 JGZTG played an analgesic role in the treatment of KOA associated with NGF-TrkA and COX-2/PGE2 signaling pathways

**FIGURE 6:**
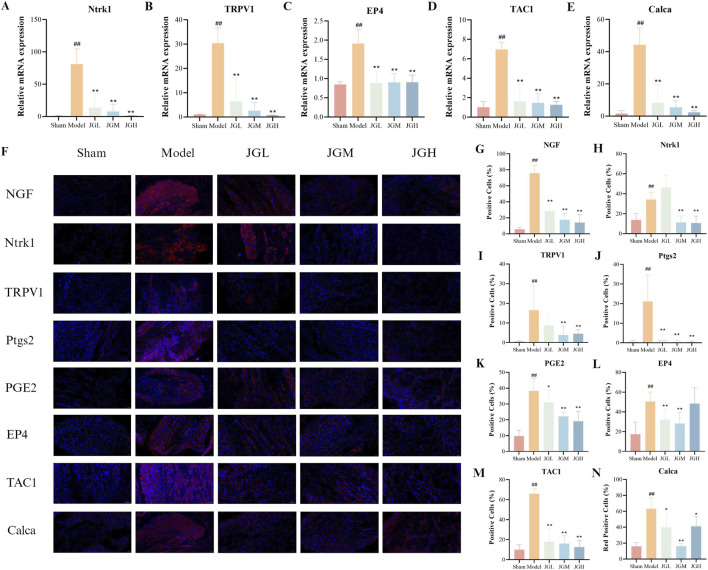
JGZTG plays an analgesic role in the treatment of KOA associated with NGF-TrkA and COX-2/PGE2 pathways. **(A–E)** RT-qPCR results of Ntrk1, TRPV1, EP4, TAC1, and Calca mRNA levels in the DRG of rats (n = 3). **(F)** Representative immunofluorescence staining images of NGF, Ntrk1, TRPV1, Ptgs2, PGE2, EP4, TAC1, and Calca in the DRG of rats (n = 3 × 3). **(G–N)** Quantitative analysis of NGF, Ntrk1, TRPV1, Ptgs2, PGE2, EP4, TAC1, and Calca immunofluorescence (n = 3 × 3). Data are presented as the means ± SD. ^##^ *P* < 0.01 vs. Sham; ^**^ *P* < 0.01 vs. Model; ^*^ *P* < 0.05 vs. Model.

Based on the findings of the expression and release of the cytokine NGF in synovial tissue, it is speculated that the NGF-TrkA pathway may be associated with the analgesic effects of JGZTG. Therefore, we explored whether the analgesic effects of JGZTG are related to the NGF-TrkA pathway by RT-qPCR and WB experiments on DRG. RT-qPCR results revealed that the relative mRNA expressions of Ntrk1, TRPV1, TAC1, and Calca were increased in the DRG of the model group compared with the sham group (^##^ *P* < 0.01). In contrast, these expressions were significantly decreased after JGZTG treatment compared with the model group (^**^ *P* < 0.01) (Figures 6A–E). IF analyses demonstrated a significant increase in the expression of NGF, Ntrk1, TRPV1, TAC1, and Calca proteins in the DRG of the model group compared with the sham group (^##^ *P* < 0.01), while treatment with JGZTG led to a notable decrease compared with the model group (^**^ *P* < 0.01,^*^ *P* < 0.05) (Figures 6F–N). Additionally, we investigated the effects of JGZTG on the COX-2/PGE2 pathway. COX-2 acts as a key enzymatic mediator in the biosynthesis of PGE2; the binding of PGE2 to the EP4 receptor is known to promote nociceptive signaling and exacerbate disease progression (Jiang et al., 2022; Peng et al., 2025). Experimental studies have revealed that JGZTG significantly inhibits the overexpression of Ptgs2, PGE2, and EP4 in KOA rats, thereby exerting analgesic effects. In conclusion, JGZTG may decrease the sensitivity to pain via the NGF-TrkA and COX-2/PGE2 pathways.

## 4 Discussion

KOA is a chronic inflammatory joint disease characterized by articular cartilage damage, subchondral bone sclerosis, peripheral bone hyperplasia, and inflammatory hyperplastic changes in synovium. The treatment of KOA is mainly focused on anti-inflammatories and relieving pain, but single-target drugs, including NSAIDs, cannot fundamentally prevent the development of KOA and have toxic side effects. TCM, characterised by its multi-component, multi-target and multi-pathway properties, has unique advantages in the treatment of KOA, and JGZTG is a gel preparation with a certain history of clinical application and definite therapeutic effects (Liu, 2012; Peng et al., 2023; Zhao et al., 2020; Zheng et al., 2022). Although the pharmacological effects and clinical efficacy of JGZTG in treating KOA have been confirmed, there is still limited specific investigation into its impact on KOA and the associated signaling pathways.

As a chronic degenerative disorder affecting the entire joint, KOA primarily manifests as persistent pain accompanied by non-specific inflammation. The synovium serves as the boundary between the internal structures of a joint and adjacent soft tissues, and research has shown that synovial lesions can promote the progression of KOA (Loeser et al., 2012; Mathiessen and Conaghan, 2017; Scanzello and Goldring, 2012). Besides, cartilage collagen plays a crucial role in maintaining the stability of the internal environment. During the development of KOA, the degradation and recombination of cartilage collagen are one of the important reasons for cartilage destruction (Li et al., 2023; Rahaman et al., 2025; Torga et al., 2024).

Our results showed that JGZTG could not only increase the joint pressure pain threshold and foot weight-bearing capacity of KOA model rats, but also decrease the pro-inflammatory cytokines levels of TNF-α, IL-6, IL-1β, and PGE2 in serum and joint lavage fluid of KOA model rats. Besides, JGZTG could significantly reduce the morphological damage of knee joint tissue, the pathological injury of joint synovial tissue, the pathological injury of knee cartilage, and the loss of cartilage collagen of the KOA model rats. These results indicated that JGZTG may achieve the therapeutic effect in KOA by inhibiting inflammatory responses, reducing collagen damage, alleviating pain, and reducing damage to the synovial membrane and cartilage of joints.

**FIGURE 7:**
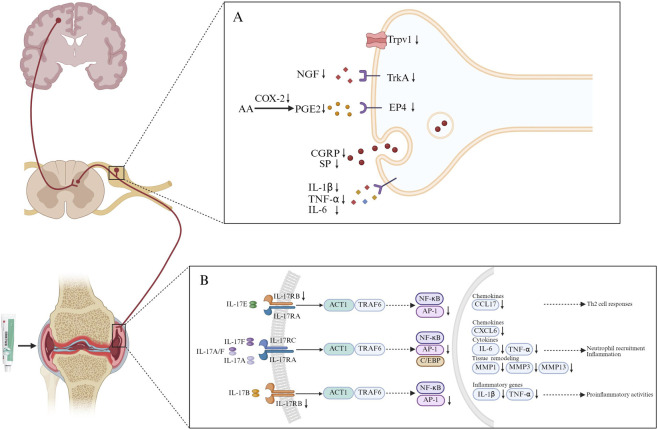
Diagram of the action mechanism of JGZTG. **(A)** JGZTG could potentially inhibit the activation of the NGF-TrkA and COX-2/PGE2 signaling pathway, mitigate the production of CGRP and SP, and Inhibit the opening of TRPV1 ion channels, thereby affirming its potent analgesic effect by lowering the sensitivity of peripheral and central nerves. **(B)** JGZTG could suppress the expression and release of chemokines (CCL17 and CXCL6), inflammatory cytokines (IL-6, TNF-α, IL-1β), and MMPs (MMP1, MMP3, and MMP13) linked to the IL-17 signaling pathway, thereby alleviating synovial inflammation in KOA. Moreover, inflammation and pain are closely interrelated in KOA. JGZTG could inhibit pain-related pathways and cytokine activation by suppressing inflammatory cytokines (IL-6, TNF-α, IL-1β) and pro-inflammatory modulators (NGF) produced during synovial inflammation.

The transcriptomic analysis result of synovium revealed that the therapeutic mechanism of JGZTG in treating KOA may be related to the IL-17 signal pathway. The IL-17 signaling pathway is a crucial pro-inflammatory pathway in the immune system, primarily mediated by the IL-17 cytokine family and its receptors (Matsuzaki and Umemura, 2018; Saima et al., 2023). The classic pathway is triggered when IL-17A or IL-17F (or their heterodimers) binds to the interleukin 17 receptor (IL-17R) A/IL-17RC complex on the cell surface. Moreover, IL-17RB, as a member of the IL-17 receptor family, can be activated by IL-17B or IL-17E. In addition to this classical axis, other family members such as IL-17B, a member of the IL-17 cytokine family with 29% homology to IL-17A (Bie et al., 2017), are also involved in inflammation. Its receptor IL-17RB, activated by IL-17B or IL-17E, is highly expressed in synovial fibroblasts in arthritis (Kouri et al., 2013). IL-17B may contribute to chronic disease and tissue destruction in KOA by promoting neutrophil proliferation and survival, while also enhancing TNF-α-induced cytokine/chemokine production that regulates immune cell trafficking and neutrophil homeostasis in inflamed tissues (Kouri et al., 2013). Given this, we identified IL-17RB as a potential core target for JGZTG in KOA, as transcriptomic analysis revealed its specific expression but not that of IL-17RA, leading to our selection of IL-17RB for further investigation. The binding of IL-17 ligand and receptor recruits tumor necrosis factor receptor-associated factor (TRAF6) via actin-related gene 1 (ACT1), and subsequently activates AP-1/NF-κB transcriptional network, thereby triggering downstream inflammatory and immune responses. This experimental study found that JGZTG can reduce the overexpression of AP-1 in the model group by acting on IL-17RB.AP-1 (with FOSB as its core component) serves as a key gene in the IL-17 signaling pathway. Upon activation, FOSB mediates transcriptional regulation that promotes the secretion of downstream pro-inflammatory mediators, chemokines, and MMPs, thereby driving synovial inflammation and facilitating cartilage degradation (Liu et al., 2022; Xu et al., 2024). CCL17 (also known as thymic and activator-regulated chemokine, TARC) is a member of the CC chemokine family and a key mediator of type II immune responses, which can be activated by AP-1. It has been reported that CCL17 expression is increased in the synovial tissue of patients with rheumatoid arthritis (Moret et al., 2013). Moreover, as a member of the CXC chemokine family, CXCL6 (also known as granulocyte chemotactic protein-2, GCP-2) primarily mediates neutrophil recruitment and activation. Synovial fibroblasts in arthritis patients can secrete CXCL6, recruit neutrophils, release MMPs, and cause joint cartilage destruction (Scanzello and Goldring, 2012). Additionally, as the most significant factor in cartilage damage, MMPs are primarily responsible for degrading components of the extracellular matrix (ECM). MMP1 and MMP13 are collagenases that primarily degrade fibrous collagen by cleaving the collagen triple helix region. MMP-3, as a matrix-dissolving enzyme, primarily degrades proteoglycans, laminin, and fibronectin. In patients with arthritis, the expression of MMP1, MMP3, and MMP13 in synovial tissue is abnormally elevated, leading to degradation of the extracellular matrix and subsequent cartilage destruction (Milaras et al., 2021; Mukherjee and Das, 2024; Murphy et al., 2002; Wassilew et al., 2010) (Figure 7B).

JGZTG appeared to influence downstream signaling within the IL-17 pathway mainly by suppressing the secretion of pro-inflammatory cytokines such as TNF-α, IL-6, and IL-1β in both serum and joint lavage fluid. It also downregulated the expression of MMPs (MMP1b, MMP3, and MMP13) as well as chemokines, including CXCL6 and CCL17. These findings suggest that JGZTG may ameliorate synovial inflammation and inhibit cartilage degradation, potentially through suppression of the IL-17 signaling pathway (Figure 7B).

Furthermore, experimental studies have demonstrated that JGZTG reduces the overexpression of NGF in synovial tissue. NGF is a pro-inflammatory cytokine that can be upregulated by cytokines such as TNF-α and IL-1β and is produced by non-neuronal cells, including synovial fibroblasts and macrophages. Synovial NGF released into the joint microenvironment acts in a paracrine manner on its high-affinity receptor TrkA (encoded by Ntrk1), which is expressed on peptidergic sensory nerve terminals innervating the synovium. This interaction initiates peripheral sensitization and subsequently facilitates the transmission of pain signals to peripheral neural tissues such as the DRG.

The synovial NGF-TrkA signaling complex undergoes retrograde transport to the DRG, where it promotes *de novo* NGF synthesis. NGF binding to TrkA receptors activates TRPV1 ion channels, resulting in increased neuronal excitability and peripheral sensitization (Liu et al., 2024). Moreover, NGF upregulates the expression of neurotransmitters such as calcitonin gene-related peptide (CGRP; encoded by Calca) (Jiang et al., 2024), substance P (SP; encoded by TAC1) (Shang et al., 2017), and brain-derived neurotrophic factor (BDNF) (I et al., 2022), all of which contribute to central sensitization (Figure 7A).

The NGF-TrkA signaling axis represents a core molecular mechanism underlying pain in knee osteoarthritis (KOA) (Denk et al., 2017; Rodriguez-Palma and Khanna, 2025). Our data indicate that JGZTG suppresses NGF synthesis at both synovial and DRG sources. The reduction in synovial NGF attenuates peripheral afferent drive, whereas the reduction in DRG NGF dampens central sensitization by limiting neuropeptide release at spinal terminals. Accordingly, the analgesic profile of JGZTG is best characterized as peripherally dominant but spinally reinforced.

In addition, the suppression of PGE2 release in the plasma and synovial fluid of model rats by JGZTG indicates that its mechanism of action likely involves the COX-2/PGE2 pathway. PGE2 is a common lipid mediator of inflammatory pain. Inflammation in peripheral tissues induces the expression of COX-2 (encoded by Ptgs2) in inflammatory tissues and the central nervous system. As a key enzyme in PGE2 production, COX-2 promotes increased PGE2 production (Cheng et al., 2021). In the central nervous system, PGE2 can activate Prostaglandin E receptor 4(Ptger4) (EP4)to release neurotransmitters such as glutamate, thereby causing central sensitization. Increased release of PGE2 also provides a sensitizing stimulus for TRPV1, enabling nerve fibers to transmit information about noxious stimuli to the thalamus and spinal cord (Ma et al., 2019) (Figure 7A). The researchers demonstrated that EP4, is the primary receptor involved in joint inflammation and pain in rodent models of rheumatoid and osteoarthritis (Clark et al., 2008).

In the experiment, it was found that JGZTG can reverse the overexpression of NGF, Ntrk1, TRPV1, TAC1, Calca, PGE2, Ptgs2, and EP4 in dorsal root ganglion tissue from KOA model rats (Figure 6), thereby lowering the sensitivity of peripheral and central nerves and achieving a pain-relieving effect. These results offer further evidence that JGZTG appears to influence the NGF-TrkA and COX-2/PGE2 pathways.

The findings of this study indicate that JGZTG exerts anti-inflammatory and analgesic effects through multiple combined pathways, inhibiting synovial inflammation, improving cartilage damage, and delaying the progression of KOA. However, the present study has certain limitations. Firstly, due to the limited sample size of animal tissue, validation of existing pathways is insufficient. Further validation may be conducted with additional genes and proteins in subsequent studies. Secondly, the present study did not employ pathway-specific inhibitors or neutralizing antibodies; therefore, whether suppression of IL-17, NGF-TrkA, and COX-2/PGE2 signaling is causally required for the effects of JGZTG remains to be definitively established. Moreover, this paper primarily focuses on synovial inflammation and pain, lacking a more in-depth exploration of cartilage damage. The mechanism of action of JGZTG in treating KOA remains to be further explored.

## 5 Conclusion

In conclusion, this study shows that JGZTG has a definite therapeutic effect on a rat model of KOA established by surgery, including the reduction of inflammatory factor levels, the increase of weight bearing on the right foot, and the increase of pain threshold, the improvement of joint morphology score, the alleviation of synovial and cartilage histopathological lesions, and the reduction of cartilage collagen loss. The results integrating transcriptomic analysis, RT-qPCR, IF, and WB revealed that JGZTG may influence IL-17, NGF-TrkA, and COX-2/PGE2 pathways, which are crucial in anti-inflammation and pain relief. Compared to conventional NSAIDs, JGZTG offers multi-target regulation and a superior safety profile. This study provides a scientific basis for exploring the potential mechanism of JGZTG in the treatment of KOA.

## Data Availability

The dataset presented in this study is available in the GEO data repository, accession number GSE309646.

## References

[B1] Ashraf S. Mapp P. I. Shahtaheri S. M. Walsh D. A. (2018). Effects of carrageenan induced synovitis on joint damage and pain in a rat model of knee osteoarthritis. Osteoarthr. Cartil. 26 (10), 1369–1378. 10.1016/j.joca.2018.07.001 30031926

[B53] Bie Q. Jin C. Zhang B. Dong H. (2017). A new area of study in the IL-17 family. Mol. Immunol. 90, 50–56. 10.1016/j.molimm.2017.07.004 28704706

[B2] Cheng H. Huang H. Guo Z. Chang Y. Li Z. (2021). Role of prostaglandin E2 in tissue repair and regeneration. Theranostics 11 (18), 8836–8854. 10.7150/thno.63396 34522214 PMC8419039

[B3] Clark P. Rowland S. E. Denis D. Mathieu M.-C. Stocco R. Poirier H. (2008). MF498 [N-{[4-(5,9-Diethoxy-6-oxo-6,8-dihydro-7 H-pyrrolo[3,4-g]quinolin-7-yl)-3-methylbenzyl]sulfonyl}-2-(2-methoxyphenyl)acetamide], a Selective E Prostanoid Receptor 4 Antagonist, Relieves Joint Inflammation and Pain in Rodent Models of Rheumatoid and Osteoarthritis. J. Pharmacol. Exp. Ther. 325 (2), 425–434. 10.1124/jpet.107.134510 18287210

[B4] Dantas L. O. Salvini T. d. F. McAlindon T. E. (2021). Knee osteoarthritis: key treatments and implications for physical therapy. Braz. J. Phys. Ther. 25 (2), 135–146. 10.1016/j.bjpt.2020.08.004 33262080 PMC7990728

[B5] Denk F. Bennett D. L. McMahon S. B. (2017). Nerve growth factor and pain mechanisms. Annu. Rev. Neurosci. 40, 307–325. 10.1146/annurev-neuro-072116-031121 28441116

[B6] Du X. Liu Z. Y. Tao X. X. Mei Y. L. Zhou D. Q. Cheng K. (2023). Research progress on the pathogenesis of knee osteoarthritis. Orthop. Surg. 15 (9), 2213–2224. 10.1111/os.13809 37435789 PMC10475681

[B7] Fu J. Zhang H. T. Wang X. N. Hu J. H. (2020). Volatile components in Jingu Zhitong gel based on GC-MS. Cent. South Pharm. 18 (12), 2050–2053. 10.7539/j.issn.1672-2981.2020.12.022

[B8] Gahbauer S. DeLeon C. Braz J. M. Craik V. Kang H. J. Wan X. (2023). Docking for EP4R antagonists active against inflammatory pain. Nat. Commun. 14 (1), 8067. 10.1038/s41467-023-43506-6 38057319 PMC10700596

[B9] Hunter D. J. Bierma-Zeinstra S. (2019). Osteoarthritis. Lancet 393 (10182), 1745–1759. 10.1016/S0140-6736(19)30417-9 31034380

[B10] InSung O. S. Kc R. Singh G. Das V. Ma K. Li X. (2022). Sensory neuron-specific deletion of tropomyosin receptor kinase A (TrkA) in mice abolishes osteoarthritis (OA) pain *via* NGF/TrkA intervention of peripheral sensitization. Int. J. Mol. Sci. 23 (20), 12076. 10.3390/ijms232012076 36292950 PMC9602682

[B11] Jiang W. Jin Y. Zhang S. Ding Y. Huo K. Yang J. (2022). PGE2 activates EP4 in subchondral bone osteoclasts to regulate osteoarthritis. Bone Res. 10 (1), 27. 10.1038/s41413-022-00201-4 35260562 PMC8904489

[B12] Jiang X. Guo Y. Fang M. Wang X. Zhang B. Song Y. (2024). Suppression of CGRP and TRPV1 expression in dorsal root ganglia of knee osteoarthritis rats by huojing decoction *via* TrkA/MKK3/6/p38 pathway. J. Inflamm. Res. 17, 5311–5326. 10.2147/JIR.S472341 39157588 PMC11330260

[B13] Kimourtzis G. Rangwani N. Jenkins B. J. Jani S. McNaughton P. A. Raouf R. (2024). Prostaglandin E2 depolarises sensory axons *in vitro* in an ANO1 and Nav1.8 dependent manner. Sci. Rep. 14 (1), 17360. 10.1038/s41598-024-67793-1 39075089 PMC11286870

[B14] Kouri V. P. Olkkonen J. Ainola M. Li T. F. Björkman L. Konttinen Y. T. (2013). Neutrophils produce interleukin-17B in rheumatoid synovial tissue. Rheumatology 53 (1), 39–47. 10.1093/rheumatology/ket309 24056520

[B15] Koyama T. Uchida K. Fukushima K. Ohashi Y. Uchiyama K. Inoue G. (2021). Elevated levels of TNF-alpha, IL-1beta and IL-6 in the synovial tissue of patients with labral tear: a comparative study with hip osteoarthritis. BMC Musculoskelet. Disord. 22 (1), 33. 10.1186/s12891-020-03888-w 33407301 PMC7788943

[B16] Li Y. Hou Y. Sun J. Wei J. Chai Y. Guo M. (2023). Therapeutic effect of acupotomy at Sanheyang for cartilage collagen damage in moderate knee osteoarthritis: a rabbit model. J. Inflamm. Res. 16, 2241–2254. 10.2147/JIR.S400956 37256203 PMC10225278

[B17] Li X. Zhou Y. Chen X. F. Wang H. J. Yang S. Yang J. (2024). Semi-synthetic chondroitin sulfate CS-semi5 upregulates miR-122-5p, conferring a therapeutic effect on osteoarthritis *via* the p38/MMP13 pathway. Acta Pharm. Sin. B 14 (8), 3528–3542. 10.1016/j.apsb.2024.05.016 39220883 PMC11365380

[B18] Liu X. (2012). “Clinical observation of Tongning gel in treating knee osteoarthritis (kidney deficiency and tendon vessel stagnation pattern),”. Master, Hunan University of Chinese Medicine. China National Knowledge Infrastructure. Available online at: kns.cnki.net/kcms2/article/abstract?v=d7HKHDfP4kd_xXBl72_dhGJp35t10sYNi5247KxEhRJ8kOZa33zorfxacKqa4tQv-Xmnb9kUoTaGiutnWOrGvEMQ9UNDC-ackIW-7HonJjpuY6xIuCajWIuRNCx_vokztfFssKrlWWMY7MiAERWuMlj-Mli2w4HTCuKRu88NJjoMwhFyR1ZPqf2jak4R2-Ww&uniplatform=NZKPT&language=CHS (Accessed August 6, 2025).

[B19] Liu G. He G. Zhang J. Zhang Z. Wang L. (2022). Identification of SCRG1 as a Potential Therapeutic Target for Human Synovial Inflammation. Front. Immunol. 13, 893301. 10.3389/fimmu.2022.893301 35720295 PMC9204521

[B20] Liu Z. X. Li M. C. Zhang L. Shi X. Q. Liao T. Y. Jie L. S. (2024). NGF signaling exacerbates KOA peripheral hyperalgesia *via* the increased TRPV1-Labeled synovial sensory innervation in KOA rats. Pain Res. Manag. 2024, 1552594. 10.1155/2024/1552594 38410126 PMC10896652

[B21] Liu Z. X. Song S. Y. Zhang Q. Y. Wang D. P. (2025). Research progress on treatment measures for joint function in non-surgical patients with knee osteoarthritis. J. Orthop. 64, 64–67. 10.1016/j.jor.2024.11.023 39691640 PMC11648640

[B22] Loeser R. F. Goldring S. R. Scanzello C. R. Goldring M. B. (2012). Osteoarthritis: a disease of the joint as an organ. Arthritis Rheum. 64 (6), 1697–1707. 10.1002/art.34453 22392533 PMC3366018

[B23] Lv K. H. Hu Y. M. Qian M. Y. Li L. Liu W. J. Cao L. (2024). Chemical composition analysis of Jingu Zhitong gel. Chin. Tradit. Pat. Med. 46 (03), 1025–1034. 10.3969/j.issn.1001-1528.2024.03.050

[B24] Ma W. Li L. Xing S. (2019). PGE2/EP4 receptor and TRPV1 channel are involved in repeated restraint stress-induced prolongation of sensitization pain evoked by subsequent PGE2 challenge. Brain Res. 1721, 146335. 10.1016/j.brainres.2019.146335 31302096

[B25] Mathiessen A. Conaghan P. G. (2017). Synovitis in osteoarthritis: current understanding with therapeutic implications. Arthritis Res. Ther. 19 (1), 18. 10.1186/s13075-017-1229-9 28148295 PMC5289060

[B26] Matsuzaki G. Umemura M. (2018). Interleukin-17 family cytokines in protective immunity against infections: role of hematopoietic cell-derived and non-hematopoietic cell-derived interleukin-17s. Microbiol. Immunol. 62 (1), 1–13. 10.1111/1348-0421.12560 29205464

[B27] Milaras C. Lepetsos P. Dafou D. Potoupnis M. Tsiridis E. (2021). Association of matrix metalloproteinase (MMP) gene polymorphisms with knee osteoarthritis: a review of the literature. Cureus 13 (10), e18607. 10.7759/cureus.18607 34765365 PMC8572546

[B28] Moret F. M. Hack C. E. van der Wurff-Jacobs K. M. G. de Jager W. Radstake T. R. D. J. Lafeber F. P. J. G. (2013). Intra-articular CD1c-expressing myeloid dendritic cells from rheumatoid arthritis patients express a unique set of T cell-attracting chemokines and spontaneously induce Th1, Th17 and Th2 cell activity. Arthritis Res. Ther. 15 (5), R155. 10.1186/ar4338 24286358 PMC3979121

[B29] Mukherjee A. Das B. (2024). The role of inflammatory mediators and matrix metalloproteinases (MMPs) in the progression of osteoarthritis. Biomater. Biosyst. 13, 100090. 10.1016/j.bbiosy.2024.100090 38440290 PMC10910010

[B30] Murphy G. Knäuper V. Atkinson S. Butler G. English W. Hutton M. (2002). Matrix metalloproteinases in arthritic disease. Arthritis Res. Ther. 4 (3), S39–S49. 10.1186/ar572 12110122 PMC3240148

[B31] Orth P. Madry H. (2015). Complex and elementary histological scoring systems for articular cartilage repair. Histol. Histopathol. 30 (8), 911–919. 10.14670/HH-11-620 25876650

[B32] Ostojic M. Ostojić M. Prlić J. Soljic V. (2019). Correlation of anxiety and chronic pain to grade of synovitis in patients with knee osteoarthritis. Psychiatr. Danub. 31 (Suppl. 1), 126–130. 30946731

[B33] Peng H. Y. Liu J. Li L. Su Z. Z. Zhang X. Z. Cao L. (2023). Mechanism of Jingu Zhitong gel in treatment of knee osteoarthritis based on NLRP3/IL-1β pathway. Chin. Traditional Herb. Drugs 54 (08), 2502–2508. 10.7501/j.issn.0253-2670.2023.08.017

[B34] Peng P. Zheng W. Liu Y. Huang J. Zhang B. Shen J. (2025). Imrecoxib attenuates osteoarthritis by modulating synovial macrophage polarization through inactivating COX-2/PGE2 signaling pathway. Front. Bioeng. Biotechnol. 13, 1526092. 10.3389/fbioe.2025.1526092 40357333 PMC12066670

[B35] Rahaman S. N. Lishadevi M. Anandasadagopan S. K. (2025). Unraveling the molecular mechanisms of osteoarthritis: the potential of polyphenols as therapeutic agents. Phytother. Res. 39 (5), 2038–2071. 10.1002/ptr.8455 40044420

[B36] Richard M. J. Driban J. B. McAlindon T. E. (2023). Pharmaceutical treatment of osteoarthritis. Osteoarthr. Cartil. 31 (4), 458–466. 10.1016/j.joca.2022.11.005 36414224

[B37] Rodriguez-Palma E. J. Khanna R. (2025). Decoding chronic pain in OA *via* NGF-TrkA signaling. Osteoarthr. Cartil. 33 (10), 1159–1161. 10.1016/j.joca.2025.07.002 40645556

[B38] Saima A. Farhin Muntaha T. Mohammad Nazmul I. Abdur R. Saikat M. Talha Bin E. (2023). Role of Th17 and IL-17 cytokines on inflammatory and auto-immune diseases. Curr. Pharm. Des. 29 (26), 2078–2090. 10.2174/1381612829666230904150808 37670700

[B39] Sanchez-Lopez E. Coras R. Torres A. Lane N. E. Guma M. (2022). Synovial inflammation in osteoarthritis progression. Nat. Rev. Rheumatol. 18 (5), 258–275. 10.1038/s41584-022-00749-9 35165404 PMC9050956

[B40] Scanzello C. R. Goldring S. R. (2012). The role of synovitis in osteoarthritis pathogenesis. Bone 51 (2), 249–257. 10.1016/j.bone.2012.02.012 22387238 PMC3372675

[B41] Shang X. Wang Z. Tao H. (2017). Mechanism and therapeutic effectiveness of nerve growth factor in osteoarthritis pain. Ther. Clin. Risk Manag. 13, 951–956. 10.2147/TCRM.S139814 28814877 PMC5546917

[B42] Torga T. Suutre S. Kisand K. Aunapuu M. Arend A. (2024). Cartilage collagen neoepitope C2C expression in the articular cartilage and its relation to joint tissue damage in patients with knee osteoarthritis. Biomedicines 12 (5), 1063. 10.3390/biomedicines12051063 38791025 PMC11117959

[B43] Wang Z. Han X. Xu J. Zhang W. Patel K. Zheng J. (2025). Hypothalamus regulates anabolic metabolism of articular cartilage superficial chondrocytes through PGE2 skeletal interoception. Adv. Sci. (Weinh) 12 (19), e2501039. 10.1002/advs.202501039 40138204 PMC12097074

[B44] Wassilew G. I. Lehnigk U. Duda G. N. Taylor W. R. Matziolis G. Dynybil C. (2010). The expression of proinflammatory cytokines and matrix metalloproteinases in the synovial membranes of patients with osteoarthritis compared with traumatic knee disorders. Arthrosc. J. Arthrosc. Relat. Surg., 26(8), 1096–1104. 10.1016/j.arthro.2009.12.018 20678708

[B45] Xiao J. Zhang P. Cai F. L. Luo C. G. Pu T. Pan X. L. (2023). IL-17 in osteoarthritis: a narrative review. Open Life Sci. 18 (1), 20220747. 10.1515/biol-2022-0747 37854319 PMC10579884

[B46] Xu L. Ma J. Zhou C. Shen Z. Zhu K. Wu X. (2024). Identification of key hub genes in knee osteoarthritis through integrated bioinformatics analysis. Sci. Rep. 14 (1), 22437. 10.1038/s41598-024-73188-z 39341952 PMC11439059

[B47] Yang D. Xu K. Xu X. Xu P. (2024). Revisiting prostaglandin E2: a promising therapeutic target for osteoarthritis. Clin. Immunol. 260, 109904. 10.1016/j.clim.2024.109904 38262526

[B48] Yu Y. Mao N. Yu L. Lin F. Shi X. Lu X. (2025). YiMu-QingGong san alleviates lipopolysaccharide-induced endometritis in mice *via* inhibiting inflammation and oxidative stress through regulating macrophage polarization. J. Ethnopharmacol. 337 (Pt 3), 118992. 10.1016/j.jep.2024.118992 39454706

[B49] Zhang Z. Yuan D. Jin X. Chang W. Zhang Y. Xie W. (2025). Asperosaponin VI suppresses ferroptosis in chondrocytes and ameliorates osteoarthritis by modulating the Nrf2/GPX4/HO-1 signaling pathway. Front. Pharmacol. 16, 1539092. 10.3389/fphar.2025.1539092 40093317 PMC11906723

[B50] Zhao Y. Shen Z. B. Ge J. R. Liu W. G. Yang J. X. He C. J. (2020). Efficacy and safety of tongning gel for knee osteoarthritis: a multicentre, randomized, double-blinded, parallel, Placebo-controlled, clinical trial. Evid. Based Complement. Altern. Med. 2020, 8707256. 10.1155/2020/8707256 32595745 PMC7305543

[B51] Zheng Y. X. Ge J. R. Liu W. G. Yang J. X. He C. J. Lu M. (2022). External application of Tongning gel for treatment of mild-to-moderate knee osteoarthritis with syndromes of kidney deficiency and tendon-vessel stasis: a randomized,double-blind,placebo-controlled,multicenter clinical study. J. Trad. Chin. Orthop. Trauma 34 (06), 17–24.

[B52] Zhou G. Zhang X. Gu Z. Zhao J. Luo M. Liu J. (2023). Research progress on the treatment of knee osteoarthritis combined with osteoporosis by single-herb Chinese medicine and compound. Front. Med. (Lausanne) 10, 1254086. 10.3389/fmed.2023.1254086 37841009 PMC10568449

